# Development of a colorectal cancer diagnostic model and dietary risk assessment through gut microbiome analysis

**DOI:** 10.1038/s12276-019-0313-4

**Published:** 2019-10-03

**Authors:** Jinho Yang, Andrea McDowell, Eun Kyoung Kim, Hochan Seo, Won Hee Lee, Chang-Mo Moon, Sung-Min Kym, Dong Ho Lee, Young Soo Park, Young-Koo Jee, Yoon-Keun Kim

**Affiliations:** 1 MD Healthcare Inc., Seoul, Republic of Korea; 2 Department of Health and Safety Convergence Science, Korea University, Seoul, Republic of Korea; 3 Department of Internal Medicine, School of Medicine, Ewha Womans University, Seoul, Republic of Korea; 4 Department of Internal Medicine, Inje University Haeundae Paik Hospital, Inje University College of Medicine, Busan, Republic of Korea; 5 Department of Internal Medicine, Seoul National University Bundang Hospital, Gyeonggi-do, Republic of Korea; 6 Department of Internal Medicine, Dankook University College of Medicine, Cheonan, Republic of Korea

**Keywords:** Diagnostic markers, Cancer screening

## Abstract

Colorectal cancer (CRC) is the third most common form of cancer and poses a critical public health threat due to the global spread of westernized diets high in meat, cholesterol, and fat. Although the link between diet and colorectal cancer has been well established, the mediating role of the gut microbiota remains elusive. In this study, we sought to elucidate the connection between the gut microbiota, diet, and CRC through metagenomic analysis of bacteria isolated from the stool of CRC (*n* = 89) and healthy (*n* = 161) subjects. This analysis yielded a dozen genera that were significantly altered in CRC patients, including increased *Bacteroides*, *Fusobacterium*, *Dorea*, and *Porphyromonas* prevalence and diminished *Pseudomonas*, *Prevotella*, *Acinetobacter*, and *Catenibacterium* carriage. Based on these altered genera, we developed two novel CRC diagnostic models through stepwise selection and a simplified model using two increased and two decreased genera. As both models yielded strong AUC values above 0.8, the simplified model was applied to assess diet-based CRC risk in mice. Mice fed a westernized high-fat diet (HFD) showed greater CRC risk than mice fed a regular chow diet. Furthermore, we found that nonglutinous rice, glutinous rice, and sorghum consumption reduced CRC risk in HFD-fed mice. Collectively, these findings support the critical mediating role of the gut microbiota in diet-induced CRC risk as well as the potential of dietary grain intake to reduce microbiota-associated CRC risk. Further study is required to validate the diagnostic prediction models developed in this study as well as the preventive potential of grain consumption to reduce CRC risk.

## Colorectal cancer: Factors influencing cancer risk

Tracing biomarkers of gut bacteria sheds light on how diet can affect gut composition and the risk of developing colorectal cancer (CRC). CRC is linked to diet, and scientists are examining how diet-associated changes in the gut microbiome may influence cancer risk. Yoon-Keun Kim, MD Healthcare Inc., Seoul, and Young-Koo Jee, Dankook University College of Medicine, Cheonan, South Korea, and co-workers analyzed bacterial populations in fecal samples from 89 CRC patients and 161 healthy controls. They found significant differences between patients and controls in 16 bacterial genera, these differences being potential biomarkers in diagnostic models for assessing CRC risk. The researchers used the models to determine CRC risk in mice fed diets, and found that the predicted risk was considerably reduced in mice on grain diets, especially rice or sorghum, compared to high fat diets.

## Introduction

Colorectal cancer (CRC) is the third most common cancer with the fourth highest cancer mortality in the world. Based on temporal profiles and demographic projections, CRC incidence is predicted to increase by 60% by 2030^1^. Despite global efforts to clearly define the pathogenesis of CRC, the precise etiology of CRC remains unknown. However, it has been established that CRC incidence is affected by genetic, epigenetic and environmental factors, such as diet^2^. The incidence rate of CRC has been increasing especially in developing countries. This increase may reflect a rise in the prevalence of CRC risk factors associated with westernization. The westernization of developing countries is characterized by rising unhealthy dietary habits, obesity and smoking^3,4^. The globalized spread of unhealthy, westernized diets high in red, processed meat and saturated fats is attracting concern, as it is reported that rising CRC risk is related to increased consumption of meats, animal fats, and cholesterol-rich foods^4,5^. People consuming a high-cholesterol diet have demonstrated higher CRC incidence than those who consume a low-cholesterol diet^6^. Additionally, it has been reported that native Africans with a low CRC risk and diets high in grain and vegetables are characterized by higher *Prevotella* abundance than African American counterparts with an increased risk of CRC development and diets high in red meat and fat, suggesting that gut bacteria also play a role in dietary CRC risk^7^.

Although a variety of possible mechanisms through which a high-fat diet (HFD) can lead to CRC development have been proposed, the gut microbiota has recently been revealed to be a likely mediator between diet and CRC. Over 100 trillion bacteria reside in the human gut, forming a complex community that mediates metabolism and immune functions to both directly and indirectly affect human health and disease^8^. As the impact of the gut microbiota on metabolism and disease has been uncovered, the relationship between diet, the gut microbiota and CRC has begun to emerge. An HFD is known to increase intestinal permeability, which in turn raises the level of gut microbiota-associated lipopolysaccharide (LPS)-induced local inflammation, and both phenomena that have been independently associated with CRC^9,10^. In turn, LPS has been reported to increase synthesis and serum levels of leptin, a known growth factor for colonic epithelial cells^11^. Increased serum leptin levels have been shown to be associated with both HFD-induced obesity and CRC^12^. Furthermore, leptin has been demonstrated to induce carcinogenesis by increasing the proliferation of colon cancer cells in vitro^13^. Altogether, these findings demonstrate one example of the complex network of the interactions among diet, the gut microbiota, and CRC and particularly highlight the mediating role of the gut microbiota.

Next-generation sequencing (NGS) has enabled researchers to determine the holistic bacterial community structure unique to each individual, and several studies have found that gut microbiota dysbiosis is associated with a variety of diseases, including colon cancer^14^. However, mixed results have prevented a clear consensus on the precise community dynamics between the gut microbiota and CRC. One of the most consistent bacterial groups shown to be associated with CRC carcinogenesis is *Bacteroides* spp., particularly *Bacteroides fragilis*. It has been shown that a high abundance of *Bacteroides* is associated with an increased risk of colon polyps, induces inflammation and contributes to CRC^2,15^. Overall, decreased trends in lactic acid bacteria, increased *Fusobacterium*, and altered *Bacteroides*/*Prevotella* levels have also been reported in CRC gut microbiota. While numerous factors may contribute to variations in CRC gut microbiome study outcomes, such as sample size, disease progression, age, sex, and regional dietary differences, one key confounding factor has yet to be addressed: bacterial extracellular vesicles (EVs). Bacteria release nanosized lipid bilayer-encapsulated EVs composed of proteins, lipids, DNA, RNA, lipopolysaccharides, and metabolites. Released microbiota-derived EVs interact with host cells both locally and distally and control various cellular processes by transferring their cellular components^16^. The amount and composition of secreted extracellular vesicles is not static, and we have shown through metagenomic analysis that alterations in gut microbiota EVs are associated with a variety of conditions, such as inflammatory bowel disease and tight junction permeability^17,18^. However, the impact of the diverse and dynamic composition of bacterial nucleic acids contained within microbiota-derived EVs has yet to be accounted for as a confounding factor in gut microbiota metagenomic analysis.

To elucidate the mediating role of the gut microbiota in the relationship between diet and CRC, we sought to identify significant gut microbiota alterations associated with CRC. We isolated bacteria and removed all bacterial EVs from the stool of 89 CRC patients and 161 healthy controls and performed 16s rDNA metagenomic analysis on the resulting bacterial pellet. Through this analysis, we developed two CRC diagnostic models based on stepwise selection of significantly altered gut microbiota-derived biomarkers (D1-model) and two significantly increased and two significantly decreased bacterial genera (D2-model). Furthermore, we hypothesized that key bacteria associated with CRC can be regulated by diet, providing useful biomarkers for diet-mediated CRC risk. To verify this hypothesis, we conducted an in vivo study assessing gut microbial alterations and associated CRC risk in mice fed an HFD or an HFD supplemented with a variety of grains. The results of this study contribute a promising advancement in CRC theragnostics, gut microbiota-based therapeutics, and gut microbiota metagenomic analysis methodology.

## Materials and methods

### Subjects

In total, 161 healthy people (76 males and 85 females) were enrolled from Haewoondae Baek Hospital, and 89 CRC patients (52 males and 37 females) were enrolled from Ewha Womans University Hospital and Seoul National University Bundang Hospital. The healthy subjects recruited in this study visited the hospital for a regular health screening. After completion of the checkup, we selected healthy persons for the study as healthy controls who were confirmed to have no known diseases and normal laboratory test results. The exclusion criteria for healthy controls included gut disease diagnosis, medication, and previous CRC diagnosis. Furthermore, we excluded those younger than 20-years-old, cancer patients and pregnant women. There was no significant difference in age or sex between healthy controls and CRC patients (*p* > 0.05) (Table 1**)**. The present study was approved by the Institutional Review Board of Ewha Womans University Hospital (IRB No. EUMC 2014–10–048–001), Seoul National University Bundang Hospital (B-1708/412–301) and Haewoondae Baek Hospital (IRB No. 129792–2015–064). The methods conducted in this study were in accordance with the approved guidelines, and informed consent was obtained from all subjects.

**Table 1:** Clinical subject demographic information

Group	Included samples		Enrolled samples
	*N* (Male/Female)	Age	
Control	161 (76/85)	63.7 (SD 9.0)	161
Colorectal cancer	89 (52/37)	64.3 (SD 13.5)	91
*P*-value	0.117^*^	0.7317**	

*p-value of *t* test between male and female subjects

**p-value of chi-squared test

### Mouse Model

Female C57BL/6 mice that were 6 weeks of age were purchased from Orient Bio Inc. (Seongnam, Korea). All mice were housed and maintained in standard laboratory conditions of 22 ± 2 °C and 50 ± 5% humidity under 12-hour day and night cycles throughout the course of the in vivo study.

### In vivo mouse study to evaluate the effect of grain foods

Mice were randomly divided into nine groups (*n* = 5), including a control group fed a regular chow diet (RCD). The other eight groups were fed a HFD or an HFD supplemented with either nonglutinous rice, glutinous rice, rice syrup, brown rice, sorghum, buckwheat or acorn. Mice within the RCD control group were fed regular chow containing 18% dietary fat obtained from Research Diets, Inc. (New Brunswick, NJ, USA) for 4 weeks. Mice in the HFD group were fed a 60% fat diet, while mice in the grain diet groups were fed a 60% fat diet (Research Diets, Inc.) with 2% of the appropriate grain powder administered in their drinking water. Mouse body weight and food intake were measured weekly. At the conclusion of the 4-week study period, all mice were sacrificed, and cecal fluid was collected to analyze the microbiota composition.

### Bacterial and EV isolation and DNA extraction

Human feces and mouse cecal fluid samples were filtered through a cell strainer after being diluted in 10 mL of PBS for 24 hours. EVs contained in the stool samples were isolated by centrifugation at 10,000 *×* *g* for 10 min at 4°C. After centrifugation, the resulting bacterial cell pellet and EV-containing supernatant were separated. DNA contained within the bacterial pellet and supernatant was extracted using a DNA isolation kit (PowerSoil DNA Isolation Kit, MO BIO Laboratory, CA, USA) following the standard protocol in the kit guide. The DNA extracted from the isolated bacterial cells and EVs contained in each sample was quantified using a QIAxpert system (QIAGEN, Hilden, Germany).

### Metagenomic analysis

Bacterial genomic DNA was amplified with the 16s_V3_F (5′- TCGTCGGCAGCGTCAGATGTGTATAAGAGACAGCCTACGGGNGGCWGCAG -3′) and 16s_V4_R (5′- GTCTCGTGGGCTCGGAGATGTGTATAAGAGACAGGACTACHVGGGTATCTAATCC -3′) primers specific for the V3-V4 hypervariable regions of the 16s rDNA gene. The libraries were prepared using PCR products according to the MiSeq System guide (Illumina, CA, USA) and quantified using a QIAxpert (QIAGEN). Each amplicon was then quantified, set at an equimolar ratio, pooled, and sequenced with a MiSeq (Illumina) according to the manufacturer’s recommendations.

### Analysis of the microbiota composition

Raw pyrosequencing reads obtained from the sequencer were filtered according to the barcode and primer sequences using MiSeq (Illumina). Taxonomic assignment was performed by the profiling program MDx-Pro ver.1 (MD Healthcare, Seoul, Korea) that selects high-quality sequencing reads with read lengths greater than 300 bp and Phred scores higher than 20 (>99% accuracy of base call). Operational taxonomic units (OTUs) were clustered using the sequence clustering algorithm CD-HIT. Subsequently, taxonomy assignment was carried out using UCLUST and QIIME against the 16s rDNA sequence database in Greengenes 8.15.13. Based on the sequence similarities, taxonomic assignment to the genus level was performed on all 16s rDNA sequences. The microbial composition at each taxon level was plotted in a stack bar. If clusters could not be assigned at the genus level due to lack of sequences or redundant sequences in the database, the taxon was assigned at the next highest level, as indicated in parentheses.

### Development of a CRC diagnostic model

The selection of biomarkers for inclusion in the diagnostic model was based on the relative abundances of OTUs at the genus level. We selected candidate biomarkers with *p*-values < 0.05, fold-changes greater than two-fold, and average relative abundances greater than 0.1%. For the first diagnostic model (D1-model), we included age and sex as covariates and selected biomarkers for inclusion in the model by a stepwise selection method. Akaike information criterion (AIC) was used to assess model fitness of the predictive diagnostic models using differing variables, and all candidate predictive diagnostic models were calculated using logistic regression. The second diagnostic model (D2-model) was established based on two increased and two decreased biomarkers as variables and was calculated by logistic regression. Based on the analysis of all the possible variable combinations using two increased and two decreased biomarkers, we selected the diagnostic model with the highest resulting AIC value as the simplified D2-model to be used to assess CRC risk during in vivo experimentation. Mann–Whitney statistics as an estimator of AUC and the DeLong test to test the change in AUC were used^19,20^, and 10-fold cross-validation was applied.

### Statistical analysis

To avoid potential bias caused by differing sequencing depths, samples with more than 3500 reads were rarefied to a depth of 3500 reads for subsequent analysis. Significant differences between the healthy control group and CRC patient group were determined using the *t* test for continuous variables. Additionally, the Mann–Whitney test was performed to analyze microbiome differences in vivo. Findings were considered significant if the *p*-value was less than 0.05 or the adjusted p-value (Ad. p) was less than 0.05. The alpha diversity of microbial composition was measured using the Chao1 index and rarified to compare species richness. Shannon’s index was used to measure the species diversity of samples between the healthy control group and CRC patient group. All statistical analyses were performed using R version 3.4.1.

## Results

### Fecal microbiota diversity of CRC patients vs. healthy controls

**Fig. 1:**
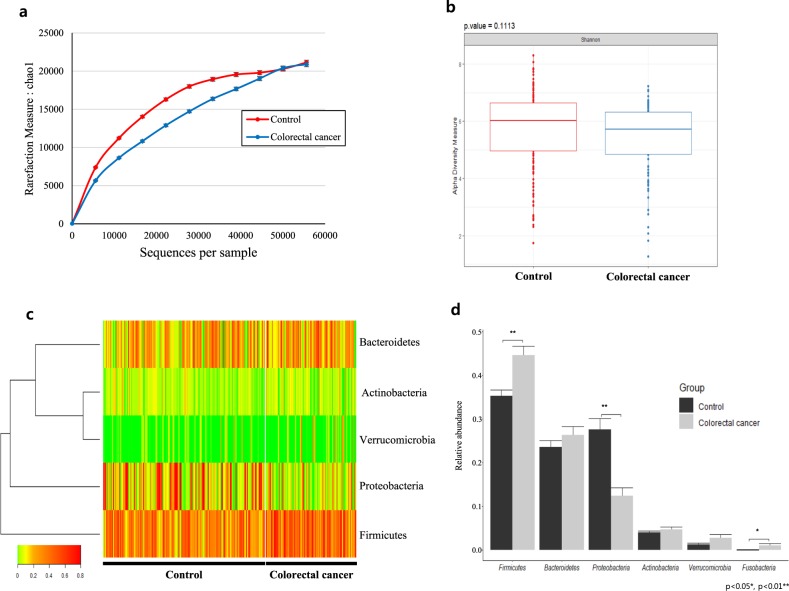
Alpha diversity and phylum-level gut microbiota composition. **a** Estimated species richness (Chao1 measure) and **b** alpha diversity defined by Shannon’s index. **c** Heatmap of the gut microbiota at the phylum level, with columns representing individual control and CRC stool samples and rows corresponding to the identified phyla. Color scale based on relative OTU abundance, and hierarchical clustering based on Euclidean distance. **d** Average relative abundance of individual phyla, with error bars representing the standard error (SE). Significance between groups assessed by a *t* test (* = Ad. *p* < 0.05, ** = Ad. *p* < 0.01)

Microbial diversity within the human fecal samples was measured using the Chao1 and Shannon diversity indexes. Through this analysis, the healthy control group showed high richness (*p* < 0.001) in both Chao1 and Shannon index diversity. While there was an observable trend of increased alpha diversity and species richness in the control group relative to those in the case group, neither Chao1 nor Shannon index measures yielded a significant difference (Figs. 1a, b). CRC patients were shown to have 1.18 times more OTU reads than the healthy control subjects, while the number of valid reads in the normal group was significantly higher than that in the colorectal group, with 58537.1 (SD 24831.5) and 50880.8 (SD 27830.7) valid reads, respectively (*p* = 0.026).

### Compositional difference of the fecal microbiota of CRC patients vs. healthy controls

**Fig. 2:**
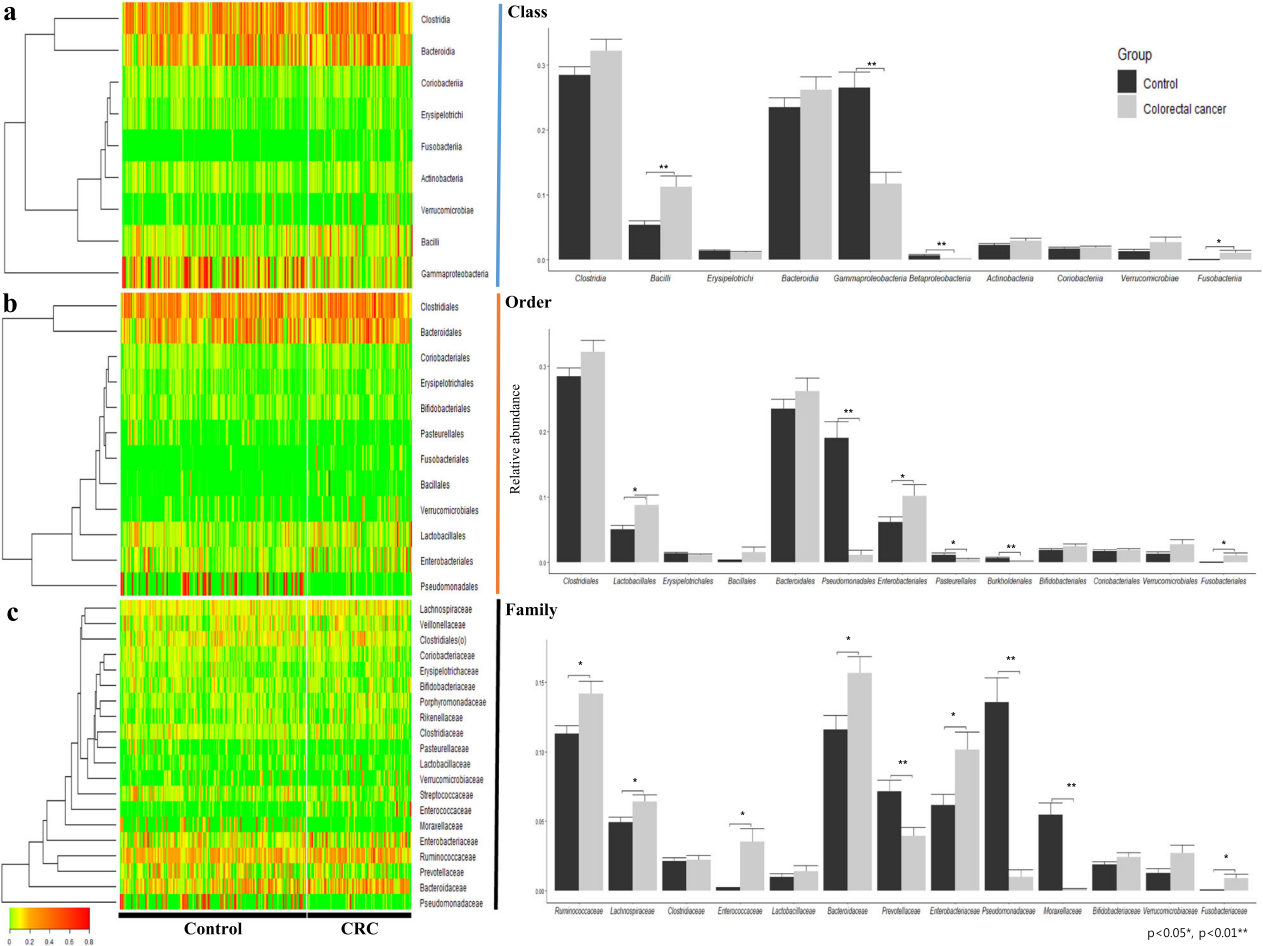
Composition of the gut microbiota at the class, order and family levels. **a** The left-side heatmap plots and hierarchical clustering dendrograms show the gut microbiota composition between individual control and CRC samples at the **a** class, **b** order, and **c** family levels. Relative abundances of individual taxa (rows) in each sample (columns) are indicated in the associated color scale. Right-side bar plots highlight the differing average relative abundance of individual key taxa between control and CRC subject stool microbiota at the **a** class, **b** order, and **c** family levels. Significant differences were calculated by a *t* test (* = Ad. *p* < 0.05, ** = Ad. *p* < 0.01)

**Fig. 3:**
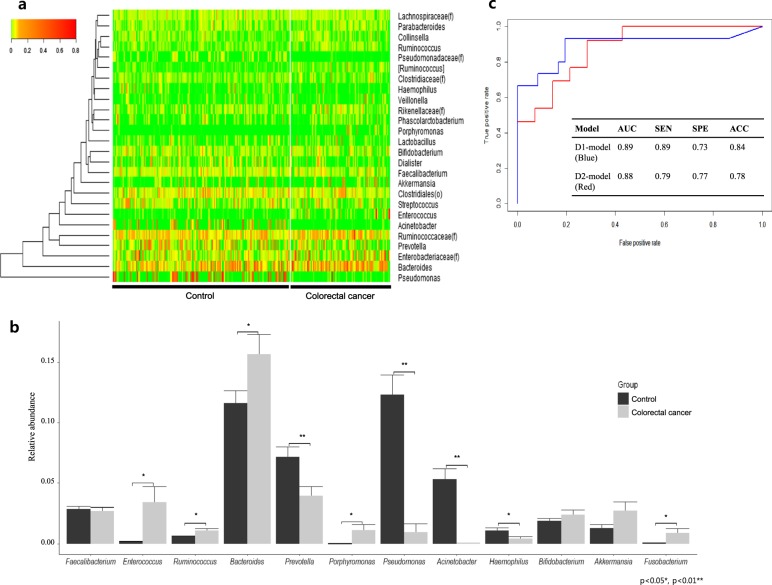
Genus level gut microbiota composition and CRC diagnostic prediction model. **a** Heatmap and clustering of individual control and CRC samples with a color scale indicating relative abundance at the genus level and hierarchical clustering measured by Euclidean distance. **b** Bar graph displaying the relative abundance of select genera and error bars showing the standard error (SE). Significance between control and CRC groups determined through Student’s *t* test (* = Ad. *p* < 0.05, ** = Ad. *p* < 0.01). **c** ROC curves of CRC diagnostic prediction models developed through stepwise selection of significantly altered genera (D1-model) and two increased and two decreased genera (D2-model). Models were validated by a 10-fold cross-validation method to assess the area under the curve (AUC), sensitivity, specificity, and accuracy of each model

Based on metagenomic analysis at the phylum level, *Firmicutes* and *Fusobacteria* were significantly increased in CRC patient samples, while Proteobacteria was significantly decreased (*p* < 0.05). In particular, Proteobacteria was vastly altered, with a 0.45-fold difference between CRC and healthy subjects (Figs. 1c, d). At the class level, carriage of Gammaproteobacteria and Betaproteobacteri*a* affiliated with Proteobacteria was significantly lower in the CRC patient group than in the healthy control group, while Bacilli and Fusobacteriia were significantly higher (*p* < 0.05) (Fig. 2a). At the order level, the case group showed significantly lower carriage than the healthy control group of Pseudomonadales, Burkholderiales, and Pasteurellales, while Fusobacteriales, Lactobacillales, and Enterobacteriales were significantly higher in the CRC group than in the healthy control group (*p* < 0.05). Although Proteobacteria was decreased overall at the phylum level, the order *Enterobacteriales* showed increased carriage in the CRC group (Fig. 2b). At the family level, carriage of Pseudomonadaceae, Moraxellaceae, Prevotellaceae, and Pasteurellaceae was significantly lower in the CRC group than in the healthy control group, while carriage of Enterococcaceae, Porphyromonadaceae, Bacteroidaceae, Enterobacteriaceae, Ruminococcaceae, and Lachnospiraceae was significantly increased in the CRC group (*p* < 0.05). Pseudomonadaceae and Moraxellaceae showed particularly dramatic fold-changes of 0.07 and 0.02, respectively (Fig. 2c). At the genus level, *Bacteroides*, *Ruminococcaceae*(f), *Enterobacteriaceae*(f), *Enterococcus*, *Ruminococcus*, *Porphyromonas*, and *[Ruminococcus]* showed a significant increase in CRC patients, while *Pseudomonas*, *Prevotella*, *Acinetobacter*, *Haemophilus*, *Pseudomonadaceae*(f) were significantly decreased (*p* < 0.05). Notably, *Porphyromonas, Enterococcus, [Ruminococcus], Acinetobacter, Pseudomonadaceae(f), Pseudomonas* and *Haemophilus* showed drastic fold changes of 85-, 20-, 4.4-, 0.01-, 0.02-, 0.08- and 0.36-fold, respectively (Figs. 3a, b).

### Diagnostic model for colorectal cancer

Bacterial biomarker candidates were selected based on three criteria: a statistically significant difference (*p* < 0.05) between the relative abundance in CRC and healthy subjects, a greater than two-fold change in relative abundance, and an average relative abundance above 0.1% at the genus level. Following those criteria, *Pseudomonas, Acinetobacter, Enterococcus, Haemophilus, [Ruminococcus], Pseudomonadaceae(f), Porphyromonas, Catenibacterium, Dorea, Fusobacterium, Erysipelotrichaceae(f), Gemellaceae(f), Cupriavidus, Peptostreptococcus, Parvimonas, Desulfovibrio*, and *Prevotella* were selected as candidate CRC biomarkers. Eight biomarker candidates, *Pseudomonadaceae*(f), *Enterococcus*, *Peptostreptococcus*, *Cupriavidus*, *Fusobacterium*, *[Ruminococcus], Desulfovibrio*, and *Erysipelotrichaceae*(f), were selected using stepwise selection with age and sex as covariates. Using these 10 variables, we created the D1 model using logistic regression with the following function:

$$S_D1 = e^{(y_D1)}/(1 + e^{(y_D1)}){\;\mathrm{with}}\,y_D1 = ax_1 + bx_2 + cx_3 + dx_4\, + ex_5 + fx_6 + gx_7 + hx_8 + ix_9 + jx_10 + k$$

In this D1-model, the values *a* to *k* are the independent parameters, and variables *x*_*1*_ *to x*_*10*_ represent age, sex, and the relative abundances of *Pseudomonadaceae(f), Enterococcus, Peptostreptococcus, Cupriavidus, Fusobacterium, [Ruminococcus], Desulfovibrio*, and *Erysipelotrichaceae(f)*, respectively. The values of these parameters are as follows: *a* is 0.06 (CI: 0.01–0.12), *b* is 1.22 (CI: 0.31–2.19), *c* is -749.7 (CI: −2679.3 to −137.9), *d* is 94.33 (CI: 49.77–201.65), *e* is 72380 (CI: 31695.5–120109.8), *f* is −5327000 (CI: −12332540 to −2361652), *g* is 409 (CI: 15.41–1520.48), *h* is 53.73 (CI: 3.17–123.70), *i* is 288.2 (CI: −39.70–855.63), *j* is 60.6 (CI: −0.31–145.80), and *k* is −6.146 (CI: −10.15 to −2.61). The D1-model test set yielded an AUC of 0.91 (SD 0.06), sensitivity of 0.85 (SD 0.14), specificity of 0.87 (SD 0.10), and accuracy of 0.86 (SD 0.06) (cut-off value of 0.51) (*p* = 0.00001) **(**Fig. 3c**)**.

In addition to the stepwise selection-based D1 model, we sought to develop a simplified diagnostic prediction model using only two increased and two decreased genera of the 17 filtered biomarkers and no clinical covariates. Sixty model variations were screened following those criteria, with 8 models yielding an AUC above 0.8. Based on this analysis, the most appropriate and relevant markers for the simplified diagnostic prediction model were determined to be *Prevotella*, *Catenibacterium*, *Dorea*, and *Porphyromonas*. The simplified D2-model constructed using these four biomarkers and a logistic regression model was created with the following function:

$$S_D2 = e^{(y_D2)}/(1 + e^{(y_D2)}){\;\mathrm{with}}\,y_D2 = ax_1 + bx_2 + cx_3 + dx_4 + e$$

In the D2-model function, *a, b, c, d*, and *e* are the independent parameters, and *x*_*1*_*, x*_*2*_*, x*_*3*_, and *x*_*4*_ represent the relative abundance of *Prevotella, Catenibacterium, Dorea*, and *Porphyromonas*, respectively. The independent parameters’ values are −4.51 (CI: −11.44–1.27) for *a*, −15.80 (CI: −60.01–7.03) for *b*, 148.00 (CI: 49.68–260.51) for *c*, 166.65 (CI: 32.47–444.20) *d*, and −1.26 (CI: −2.13 to −0.46) for *e*. The above D2-model yielded an AUC of 0.80 (SD 0.14), sensitivity of 0.79 (SD 0.17), specificity of 0.82 (SD 0.16) and accuracy of 0.80 (SD 0.12) (cut-off value 0.27), based on analysis using the test set (*p* = 0.0004) (Fig. 3c). The difference between the D1-model and D2-model was not significant (*p* = 0.858)

### Compositional difference of the cecal microbiota of mice fed an HFD vs. RCD

**Fig. 4:**
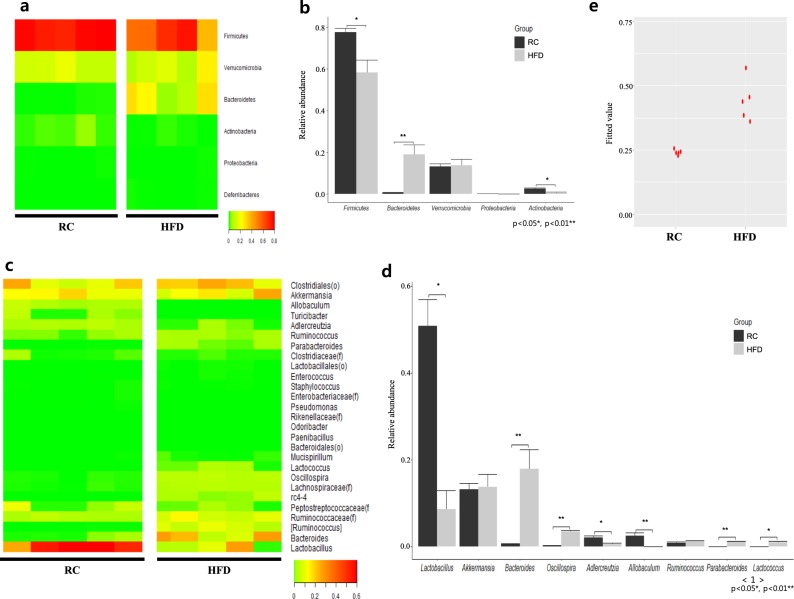
Gut microbiota composition and CRC risk differed between RCD and HFD mice. Heatmap and hierarchical clustering of gut microbiota relative abundance of individual control regular chow diet-fed (RCD) and high-fat diet-fed (HFD) mouse stool samples at the **a** phylum and **c** genus levels. The average relative abundances of individual taxa identified in RCD and HFD mouse stool at the **b** phylum and **d** genus levels. Standard errors (SEs) represented by error bars and significant differences between groups measured by the Mann–Whitney test (* = Ad. *p* < 0.05, ** = Ad. *p* < 0.01). **e** Predicted values of CRC risk in RCD and HFD mice are based on the D2 model

In contrast with the microbiota composition of CRC patient samples, *Firmicutes* was significantly decreased in HFD-fed mice (*p* < 0.05), while Proteobacteria showed no difference. Bacteroidetes showed significant enrichment, while Actinobacteria was significantly diminished in RCD-fed mice (Figs. 4a, b). At the class level, HFD-fed mice had higher carriage of Clostridia and Bacteroidia than RCD-fed mice, while Bacilli, Coriobacteriia, and Erysipelotrichi abundance was significantly greater in RCD-fed mice than in HFD-fed mice. Only Clostridia affiliated with Firmicutes was significantly increased in the HFD group (*p* < 0.05). At the order level, Clostridiales and Bacteroides abundances in HFD-fed mice were higher than in RCD-fed mice, while Lactobacillales, Coriobacteriales, Erysipelotrichales, and Turicibacterales were less prevalent in HFD-fed mice than in RCD-fed mice (*p* < 0.05). Bacteroidales, Lactobacillales, Erysipelotrichales, and Turicibacterales experienced drastic alterations, with 38.5-fold, 0.19-fold, 0.07-fold, and 0.001-fold changes, respectively. Meanwhile, at the family level, proportions of Bacteroidaceae, Ruminococcaceae, Lachnospiraceae, Peptococcaceae, and Porphyromonadaceae were significantly higher in HFD-fed mice than in RCD-fed mice, while Lactobacillaceae, Coriobacteriaceae, and Erysipelotrichaceae proportions were significantly lower (*p* < 0.05). In particular, Bacteroidaceae, Lachnospiraceae, Peptococcaceae, and Porphyromonadaceae were sharply increased in HFD-fed mice, with 38.1-fold, 29.1-fold, 242.4-fold, and 48.7-fold increases, respectively, while Erysipelotrichaceae showed a steep 0.07-fold reduction in the HFD model.

Finally, at the genus level, *Bacteroides*, *Ruminococcaceae*(f), and *[Ruminococcus]* each showed highly significant increases in HFD-fed mice. *Ruminococcus*, however, demonstrated no significant difference between mice fed an HFD or RCD and accounted for a relatively low portion of the total microbiota. Furthermore, *Oscillospira*, *Lachnospiraceae*(f), *rc4–4*, and *Parabacteroides* were significantly enriched, while *Lactobacillus*, *Adlercreutzia*, *Turicibacter*, and *Allobaculum* were significantly depleted in HFD-fed mice. *Bacteroides*, *rc4–4, [Ruminococcus]*, and *Parabacteroides* showed particularly higher carriage in HFD-fed mice than in RCD-fed mice, with 268-fold, 178-fold, 48-fold, and 38-fold increases, respectively. Meanwhile, *Turicibacter* and *Allobaculum* were extremely depleted in HFD-fed mice, with proportions of 1.3% and 2.4% in the control RCD-fed group, respectively, while possessing less than 10^–5^% of the total population in the HFD group **(**Figs. 4c, d). After applying the simplified CRC diagnostic prediction model (D2-model) to the RCD and HFD groups, the analysis yielded a fitted value of 0.24 (SD 0.01) in the control RCD group, while the HFD group showed a fitted value of 0.44 (SD 0.07) (Fig. 4e). Additionally, the result applying the prediction model showed that the AUC was 1.00.

### Grain consumption reduces CRC risk in mice

**Fig. 5:**
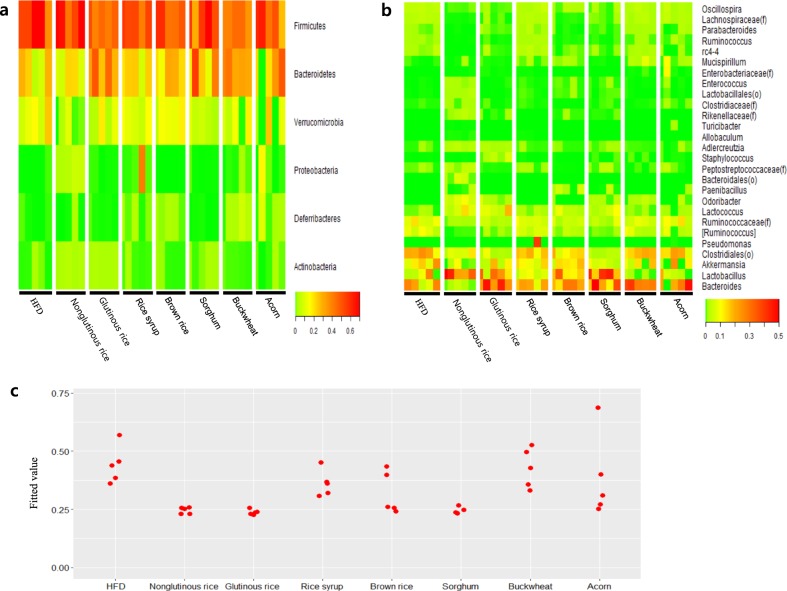
HFD mouse gut microbiota composition and associated CRC risk modulated by grain consumption. Heatmap and hierarchical clustering depicts the differential microbiome relative abundance of HFD mouse stool after consumption of seven different grains at the **a** phylum and **b** genus levels. Rows represent taxa identified in each sample, and columns represent individual samples, grouped by diet type. **c** The predicted values of CRC risk in HFD mice and HFD mice fed seven different grains using the D2 model

Microbial analysis was also conducted on the cecal content of mice after they were fed a variety of grain diets in combination with an HFD. At the phylum level, none of the grains assessed in this study were shown to significantly decrease Firmicutes, a phylum that was significantly increased in the CRC group. However, nonglutinous rice and rice syrup consumption led to a significant increase in Proteobacteria, a phylum shown to be significantly decreased in CRC patients (Fig. 5a, Table 2). At the class level, Gammaproteobacteria, a diminished class in CRC patients, was increased after consumption of rice syrup. At the order level, nonglutinous rice consumption was associated with a significant increase in the relative abundance of Pseudomonadales, a decreased order in the CRC group. At the family level, Ruminococcaceae, Lachnospiraceae, Bacteroidaceae, and Porphyromonadaceae were decreased in mice after consumption of grains compared to those in the HFD-fed mice, consistent with the differences between healthy subjects and CRC patients. Ruminococcaceae and Lachnospiraceae were significantly decreased after consumption of nonglutinous rice, glutinous rice, brown rice, and sorghum. Meanwhile, Bacteroidaceae was significantly decreased after consumption of nonglutinous rice. Porphyromonadaceae showed decreased carriage in mice after consumption of nonglutinous rice, brown rice, and sorghum. Finally, at the genus level, nonglutinous rice consumption was associated with a significant decrease in HFD-induced elevated *Bacteroides*, *Ruminococcus*, and *[Ruminococcus]* levels and further caused significant recovery of depleted *Acinetobacter*. The grain types that caused a significant decrease in *Ruminococcus* and *[Ruminococcus]* included glutinous rice, brown rice, and sorghum (Fig. 5b, Table 3). These findings were then analyzed using the D2 model to determine the CRC risk in each group. Through this analysis, the HFD group yielded a fitted value of 0.44 (SD 0.07), while the nonglutinous rice-, glutinous rice-, rice syrup-, brown rice-, sorghum-, buckwheat-, and acorn-fed groups yielded fitted values of 0.25 (SD 0.01), 0.24 (SD 0.01), 0.36 (SD 0.05), 0.32 (SD 0.08), 0.24 (SD 0.01), 0.43 (SD 0.08), and 0.38 (SD 0.16), respectively. Nonglutinous rice, glutinous rice, and sorghum were the main grain types for which consumption was shown to decrease the level of CRC risk associated with an HFD (Fig. 5c).

**Table 2:** HFD mouse microbiota composition at the phylum level before and after grain consumption

Taxon	HFD		Nonglutinous rice			Glutinous rice			Rice syrup			Brown rice			Sorghum			Buckwheat			Acorn		
	Mean	SE	Mean	SE	Ad. P	Mean	SE	Ad. P	Mean	SE	Ad. P	Mean	SE	Ad. P	Mean	SE	Ad. P	Mean	SE	Ad. P	Mean	SE	Ad. P
Firmicutes	0.583	0.061	0.618	0.053	1.000	0.451	0.021	0.333	0.521	0.026	0.760	0.512	0.034	0.7112	0.530	0.059	0.753	0.409	0.016	0.210	0.489	0.060	0.508
Bacteroidetes	0.189	0.047	0.141	0.030	0.626	0.365	0.044	0.333	0.202	0.028	1.000	0.234	0.028	0.712	0.304	0.072	0.588	0.353	0.022	0.044	0.255	0.074	0.841
Verrucomicrobia	0.137	0.030	0.029	0.018	0.102	0.078	0.029	0.333	0.113	0.016	0.760	0.123	0.021	1.000	0.060	0.014	0.190	0.091	0.029	0.669	0.081	0.044	0.464
Proteobacteria	0.001	0.001	0.041	0.009	0.102	0.002	0.001	0.333	0.085	0.072	0.175	0.000	0.000	0.052	0.002	0.001	0.588	0.004	0.002	0.349	0.036	0.030	0.404
Actinobacteria	0.006	0.002	0.021	0.003	0.102	0.017	0.002	0.333	0.005	0.001	0.760	0.006	0.001	0.816	0.009	0.001	0.588	0.004	0.001	0.690	0.007	0.003	0.841
Deferribacteres	0.002	0.001	0.004	0.003	0.897	0.002	0.001	0.753	0.011	0.004	0.175	0.011	0.002	0.052	0.001	0.000	0.588	0.030	0.011	0.116	0.028	0.018	0.333
Tenericutes	0.001	0.000	0.001	0.000	0.495	0.001	0.001	0.753	0.000	0.000	0.204	0.000	0.000	0.052	0.000	0.000	0.190	0.000	0.000	0.044	0.002	0.001	0.404

**Table 3:** HFD mouse microbiota composition at the genus level before and after grain consumption

Taxon	HFD		Nonglutinous rice			Glutinous rice			Rice syrup			Brown rice			Sorghum			Buckwheat			Acorn		
	Mean	SE	Mean	SE	Ad. P	Mean	SE	Ad. P	Mean	SE	Ad. P	Mean	SE	Ad. P	Mean	SE	Ad. P	Mean	SE	Ad. P	Mean	SE	Ad. P
*Bacteroides*	0.178	0.045	0.020	0.007	0.057	0.326	0.049	0.394	0.182	0.026	0.821	0.224	0.028	0.681	0.279	0.068	0.623	0.325	0.026	0.451	0.237	0.069	0.950
*Akkermansia*	0.137	0.030	0.029	0.018	0.090	0.078	0.029	0.394	0.113	0.016	0.821	0.123	0.021	1.000	0.060	0.014	0.138	0.091	0.029	0.762	0.081	0.044	0.649
*Lactobacillus*	0.085	0.044	0.343	0.052	0.090	0.211	0.050	0.236	0.062	0.012	0.938	0.182	0.038	0.254	0.326	0.059	0.138	0.015	0.006	0.616	0.067	0.032	0.950
*[Ruminococcus]*	0.067	0.012	0.003	0.001	0.043	0.012	0.004	0.073	0.061	0.007	0.821	0.025	0.005	0.120	0.007	0.001	0.083	0.049	0.015	0.616	0.035	0.013	0.649
*Lactococcus*	0.009	0.003	0.071	0.017	0.043	0.086	0.024	0.049	0.016	0.004	0.510	0.040	0.011	0.081	0.049	0.015	0.138	0.009	0.002	0.997	0.011	0.003	0.855
*Oscillospira*	0.035	0.002	0.005	0.001	0.043	0.007	0.002	0.049	0.030	0.005	0.597	0.017	0.003	0.060	0.012	0.004	0.083	0.024	0.004	0.616	0.023	0.006	0.649
*Parabacteroides*	0.011	0.002	0.000	0.000	0.043	0.008	0.002	0.531	0.020	0.005	0.597	0.003	0.002	0.120	0.004	0.001	0.104	0.011	0.001	1.000	0.010	0.004	0.855
*Ruminococcus*	0.012	0.001	0.000	0.000	0.043	0.002	0.001	0.049	0.018	0.002	0.377	0.002	0.000	0.060	0.002	0.001	0.083	0.008	0.002	0.616	0.010	0.004	0.649
*Adlercreutzia*	0.006	0.002	0.021	0.003	0.090	0.016	0.002	0.106	0.005	0.001	0.723	0.005	0.001	0.796	0.008	0.001	0.465	0.004	0.000	0.875	0.007	0.003	1.000
*Mucispirillum*	0.002	0.001	0.004	0.003	0.898	0.002	0.001	0.793	0.011	0.004	0.377	0.011	0.002	0.060	0.001	0.000	0.508	0.030	0.011	0.457	0.028	0.018	0.649
*Odoribacter*	0.000	0.000	0.063	0.009	0.043	0.025	0.010	0.049	0.000	0.000	–	0.003	0.002	0.548	0.018	0.004	0.083	0.017	0.006	0.254	0.008	0.006	0.649
*Pseudomonas*	0.000	0.000	0.000	0.000	0.895	0.000	0.000	0.710	0.080	0.072	1.000	0.000	0.000	0.217	0.000	0.000	0.392	0.000	0.000	1.000	0.000	0.000	0.649
*Enterococcus*	0.001	0.000	0.014	0.001	0.043	0.001	0.000	0.915	0.002	0.001	0.463	0.006	0.002	0.060	0.004	0.001	0.104	0.001	0.000	0.616	0.004	0.002	0.649
*Staphylococcus*	0.000	0.000	0.000	0.000	0.752	0.026	0.010	0.049	0.000	0.000	0.821	0.000	0.000	0.713	0.002	0.001	0.758	0.002	0.001	1.000	0.002	0.001	0.649
*Paenibacillus*	0.000	0.000	0.012	0.006	0.044	0.000	0.000	1.000	0.000	0.000	1.000	0.027	0.010	0.062	0.010	0.008	0.181	0.000	0.000	0.562	0.003	0.002	1.000
*Acinetobacter*	0.000	0.000	0.001	0.001	0.090	0.000	0.000	0.793	0.001	0.000	0.463	0.000	0.000	0.548	0.000	0.000	0.547	0.000	0.000	1.000	0.000	0.000	0.855

## Discussion

In the present study, we developed two novel CRC diagnostic models based on metagenomic analysis of stool-derived bacterial pellets separated from bacterial EVs containing bacterial DNA. As seen in Supplementary Fig. 1, the total DNA yield of bacterial EVs isolated from stool contributed to more than a quarter of the total bacterial DNA yield. This finding is critical because it reveals that more than a quarter of the bacterial sequences obtained from stool originate from bacterial EVs rather than from bacterial cells themselves. As the microbiota releases EVs differentially based on its metabolic state, proliferation, apoptosis, and community structure, the variable composition of bacterial EVs contained in stool poses a crucial confounding factor in gut microbiota metagenomic analysis^21^. To account for and eliminate potential bias caused by differential bacterial EV composition, we removed bacterial EVs contained within fecal samples via centrifugation and analyzed the resulting isolated bacterial pellet. This methodology is a distinguishing aspect of this study because gut microbiome analysis typically does not account for the potentially confounding factor of EV-originating bacterial DNA in stool. Therefore, we suggest that future gut microbiome studies consider the impact of differential microbial EV composition contained within fecal samples on microbiome profiling and take the appropriate measures to remove EVs prior to bacterial analysis.

Although we determined a multitude of taxa at different levels that were significantly altered in CRC patients (Fig. 2), we selected only those at the genera level for inclusion in the diagnostic models to enhance the model specificity and accuracy. Of the 17 significantly differing genera, 8 were selected via stepwise selection in the D1 model, in addition to age and gender. We also developed a second model, the D2 model, that included only 4 genera to offer a simplified model that is more accessible for practical diagnostic purposes. Although the D2 model using minimal biomarkers showed slightly lower accuracy, sensitivity, and specificity than the more robust D1 model, the D2 model demonstrated desirable strength as a diagnostic risk model (AUC 0.88). Overall, although the two models were similar in their CRC risk diagnosis strength, the D1-model can obtain more accurate results by utilizing both metagenomic analysis and clinical information, while the D2-model offers a more simplified option through a minimized, targeted approach. Although additional experimentation is necessary to refine the simplified, targeted D2-model, we found that four gut microbiome-derived biomarkers were sufficient to diagnose CRC risk.

Metagenomic analysis of CRC patient and healthy subject stool bacteria yielded a variety of altered genera known to be associated with CRC. A number of genera included in the D1 model, such as *Enterococcus*, *Fusobacterium*, *Peptostreptococcus* and *Desulfovibrio*, have been shown in previous studies to be enriched in CRC patients via gut microbiome metagenomic analysis^22–24^. *Fusobacterium*, in particular, has been thoroughly established as a pathogenic driver of CRC. Specifically, the overabundance of invasive *Fusobacterium nucleatum* is associated with CRC and has even been suggested to negatively impact patient outcomes^25–27^. Although it is difficult to directly establish a causal link between a single pathogenic species and CRC, possible mechanisms of carcinogenic action of invasive *Fusobacterium* spp. include induction of cascading inflammatory responses and colon tumor cell growth promotion via β-catenin activation^28^.

In the development of the targeted D2 model, two increased and two decreased bacterial genera in CRC patients were shown to yield the most accurate results: *Dorea* and *Porphyromonas* and *Catenibacterium* and *Prevotella*, respectively. *Dorea* has previously been found to be more abundant in fecal samples of CRC patients than in those of healthy controls^29^. *Dorea* spp. have the ability to adhere to cancer cells, which may confer *Dorea* a competitive advantage in the cancerous colorectal environment^30^. Meanwhile, *Porphyromonas* has been reported to be enriched in CRC patients in several studies using NGS-based gut microbiota profiling methods^22,24,31^. Furthermore, *Porphyromonas* species have been implicated as biomarkers of orodigestive cancer, as increased carriage of pathogenic, proinflammatory carcinogenic *Porphyromonas gingivalis (P. gingivalis)* as well as increased *P*. *gingivalis*-associated IgG serum antibody levels have been associated with oral, colorectal and pancreatic cancers^32^. In total, these previous findings support the association between CRC and increased abundance of *Dorea* and *Porphyromonas* in the gut and highlight the opportunistic capacity of *Dorea* spp. and the potential carcinogenic role of *Porphyromonas* spp. in CRC.

In contrast, Catenibacterium has seldom been associated with CRC, aside from a finding that *Catenibacterium* was absent in a Chinese cohort of CRC patients, which is in line with the results of this study^31^. Furthermore, we found that *Prevotella* spp. were significantly reduced in CRC patients, and multiple studies have shown increased *Prevotella* abundance in the gut microbiota and cancerous tissues of Chinese, American, and European CRC patients^31,33,34^. These findings may be explained by the connection between Tjalsma’s proposed Bacterial Driver-Carrier model of CRC and the dietary-based *Bacteroides*-*Prevotella* gradient. Tjalsma’s Bacterial Driver-Carrier model postulates that pathogenic bacterial drivers can disrupt gut microbiota balance through carcinogenic activity, such as proinflammatory signaling, secretion of genotoxic substances and other mechanisms leading to premalignant adenomas, mutations, and ultimately carcinoma development in the colorectal cavity^35^. This model posits that bacterial drivers induce gut dysbiosis and drive carcinogenic activity, enabling the enrichment of other bacterial passengers that under normal circumstances cannot effectively colonize a healthy gut. However, here, we further suggest that gut dysbiosis initiated by bacterial drivers also causes commensal bacterial passengers unsuited to the cancerous gut environment to depart the gut, based on the initial bacterial community structure.

Recently, it has been posited that the gut microbiota community structure is characterized by a *Prevotella*-*Bacteroides* gradient that enables broad classification of gut enterotypes dominated by either *Prevotella* of *Bacteroides*^36^. These gut enterotypes are significantly affected by dietary habits, as diets high in red meat and animal fat are typically associated with high *Bacteroides* and low *Prevotella* abundance, while conversely, those who consume high amounts of dietary fiber and low amounts of animal fat and protein are associated with low *Bacteroides* and high *Prevotella* abundance. This dietary-based *Prevotella*-*Bacteroides* gradient may explain our finding that *Bacteroides* was significantly increased and *Prevotella* was significantly decreased in Korean CRC patients. Previous studies have consistently reported an increased *Prevotella* abundance in CRC patients; however, these studies mostly assessed cohorts from regions known to have relatively low *Prevotella* abundance in the general population and low dietary fiber and high animal fat and protein consumption^35,37^. However, *Prevotella* is one of the most dominant genera in the Korean gut microbiota, which has been largely attributed to the relatively low consumption of animal fat and proteins and the high consumption of complex fibers and grains in the typical Korean diet^38^. Therefore, our finding of increased *Bacteroides* and decreased *Prevotella* abundance in this Korean cohort suggests a critical shift in the *Prevotella*-*Bacteroides* gradient in the cancerous gut environment. Based on the culmination of these findings, we postulate that *Prevotella* may be a bacterial passenger that departs the Korean colon as carcinogenic bacterial drivers, such as increased *Porphyromonas* and *Fusobacterium*, induce a gut environment favorable to *Bacteroides*. Furthermore, we emphasize that regional differences in diet and the *Prevotella*-*Bacteroides* gradient of the target population must be considered to fully grasp the dynamic relationship between CRC and the gut microbiome and develop accurate diagnostic prediction models.

While altered carriage of certain genera found in this study, such as *Pseudomonas, Acinetobacter, Haemophilus*, and *Parvimonas*, has been previously associated with CRC, such genera ultimately were not included in either diagnostic prediction model due to diminished model fitness^31,33^. Interestingly, in addition to the finding that *Acinetobacter* and *Pseudomonas* were severely depleted in CRC patients, conversely, we observed a general trend in healthy subjects that high *Acinetobacter* and *Pseudomonas* prevalence was associated with a sharp decrease in *Bacteroides*-*Prevotella* abundance. While discrete gut enterotypes have been established based on the dominance of either *Bacteroides* or *Prevotella* in the gut, based on our present findings, we suggest that dominance of *Acinetobacter* and *Pseudomonas* may represent a distinct third gut enterotype. In addition, as the *Bacteroides*-*Prevotella* enterotypes are strongly influenced by diet, further study is required to determine any distinguishing dietary patterns associated with *Acinetobacter-Pseudomonas* dominance, such as high grain consumption. Altogether, although our findings were generally congruent with previous studies, conflicting results may be attributed to our unique analysis method excluding DNA contributed by bacterial EVs as well as to differing regional dietary patterns in the sampled cohorts.

As dietary habits are well known to influence the risk of CRC incidence, we sought to further elucidate the relationship between the gut microbiota, diet, and CRC risk. While the impact of a westernized HFD on CRC and the gut microbiota has been well characterized, conversely, the protective effects of grain diets known to be associated with low CRC risk remain uncertain at the microbiota level. As previously discussed, populations at low risk of CRC development generally consume diets high in grain and dietary fiber and are characterized by a *Prevotella*-dominant gut enterotype. Dietary grains contain polyphenols and other antioxidant components known to promote health, reduce local inflammation in the colon and protect against colorectal cancer^39,40^. Here, we assessed the ability of seven different grains to reduce CRC risk in mice fed an HFD and found that consumption of nonglutinous rice, glutinous rice, and sorghum led to the highest reduction in CRC risk. Although the 2012 Consumer Reports claimed that concerning levels of arsenic in rice may lead to cancer risk in those who consume rice, recent epidemiologic studies have determined no cancerous risk associated with rice consumption in the United States^41^. Furthermore, previous studies have shown that Asian diets high in rice consumption were associated with reduced cancer risk^42^. Furthermore, high-performance liquid chromatography (HPLC) analysis has shown that nonglutinous rice in particular has higher phenolic content than its glutinous counterpart^43^. In the present study, while both nonglutinous and glutinous rice showed similarly low CRC risk, nonglutinous rice was especially effective in stabilizing key altered genera shown to be associated with CRC, including *Bacteroides, Lactobacillus, Ruminococcus, [Ruminococcus]*, and *Acinetobacter*. As glutinous and nonglutinous rice differ in phenolic content as well as the structure, type and distribution of starch in the vicinity of the crushed cell layer, these differences may explain the differing trends of altered genera observed in this study^44^. Sorghum, meanwhile, has previously shown tremendous anti-CRC effects by suppressing the growth and metastasis of cancerous colon epithelial cells as well as protecting against gut microbiota alterations linked to colitis, an inflammatory condition commonly associated with CRC risk^45,46^. Other grains tested in this study, such as buckwheat, rice syrup and acorn, demonstrated limited effects at offsetting HFD-induced CRC risk, highlighting the differing efficacy of different grains in reducing CRC risk. In total, these findings demonstrate the protective and preventative effect of a variety of grain-based diets on the development of CRC risk via differential stabilization of key microbiota-based biomarkers. Furthermore, as Eastern countries, such as Korea, continue to transition from traditional rice-based diets to an increasingly westernized HFD, we emphasize the importance of rice consumption in the daily diet of vulnerable populations to improve the balance of the gut microbiota and counteract the rising trend in CRC risk.

Risk assessment, early diagnosis, and prevention of disease, including for CRC, is critical for an effective reduction of mortality and increased quality of life; therefore, great effort has recently been put into advancing early cancer diagnosis, including the development of effective prediction models and in vitro diagnostics (IVD)^47,48^. Although several diagnostic models have been developed to predict CRC risk, models limited to primarily epidemiological data have shown relatively low discriminatory power, with AUCs ranging from 0.61 to 0.78^49,50^. Diagnostic models based on risk factor profiles obtained via in vitro methodologies, such as serum metabolomics, showed much higher discriminating ability, with an AUC up to 0.91; however, the high price of such IVD methodologies may prevent the widespread general use of such prediction models^51^. Thus, we aimed to develop a cost-effective diagnostic model that maintained the high discriminatory power expected from IVD methodologies by utilizing microbiome analysis. The simplified D2 model developed in the present study required only four key bacterial taxa to maintain an AUC of 0.88, showing the high discriminatory power contained within the gut microbiota to assess CRC risk. While this study strongly supports the potency of gut microbiota-based IVD, further clinical studies are necessary to confirm the efficacy of our diagnostic models and the effect of grain consumption on CRC patients at varying stages of disease progression. Unfortunately, we could not include patient BMI and smoking history as covariates in this study because we were unable to obtain sufficient information on those variables from the subjects utilized for diagnostic model development. We are continuously collecting more stool samples from both healthy subjects and CRC patients with a focus on obtaining as much thorough clinical information and background as possible for inclusion of more covariables in future microbiome-based disease diagnostic model development.

In conclusion, our results highlight the important mediating role of the gut microbiota in the relationship between diet and CRC. First, we identified 16 significantly altered genera with potential as biomarkers of CRC risk and developed two novel gut microbiota-based CRC risk assessment models. We used the simplified D2 model to assess the role of diet in CRC risk and found that an HFD increased CRC risk in mice. Next, we compared the effect of an HFD and a variety of grain-based diets on microbiota composition and subsequent CRC risk in mice and found that nonglutinous rice, glutinous rice, and sorghum consumption vastly reduced CRC risk. Taken together, these results suggest the utility and validity of gut microbiota-based CRC risk assessment as well as dietary-based prevention to reduce CRC risk in the development of an effective CRC theragnostic strategy.

## Supplementary information

Supplementary Information
