# Supplementary figures and images for: Development of a colorectal cancer diagnostic model and dietary risk assessment through gut microbiome analysis
Source: Exp Mol Med. 2019 Oct 3;51(10):117. doi: 10.1038/s12276-019-0313-4 (PMC6802675; doi:10.1038/s12276-019-0313-4)

# Supplementary Figure 1

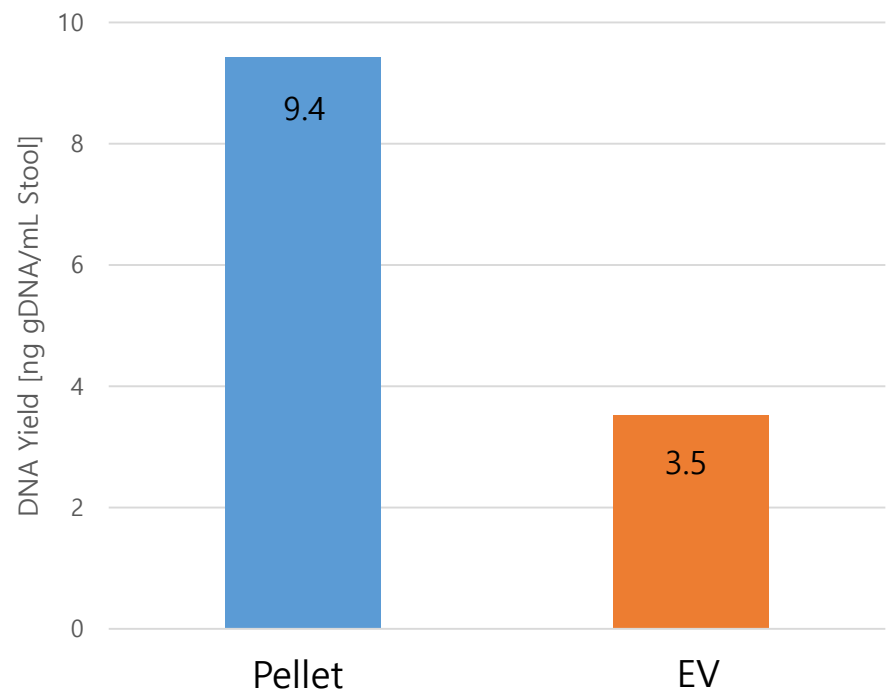

Supplement: Supplementary file 1 — Supplementary Information [file 12276_2019_313_MOESM1_ESM.pdf]
